# Structural and biochemical data of *Trichoderma harzianum* GH1 β-glucosidases

**DOI:** 10.1016/j.dib.2017.09.044

**Published:** 2017-09-22

**Authors:** Renata N. Florindo, Valquiria P. Souza, Hemily S. Mutti, Lívia R. Manzine Margarido, Cesar Camilo, Sandro R. Marana, Igor Polikarpov, Alessandro S. Nascimento

**Affiliations:** a Instituto de Física de São Carlos, Universidade de São Paulo, Av. Trabalhador Saocarlense, 400, Centro, Sao Carlos, SP 13566-590, Brazil; b Departamento de Bioquímica, Instituto de Química, Universidade de São Paulo, São Paulo, SP, Brazil

## Abstract

Here the statistics concerning X-ray data processing and structure refinement are given, together with the substrate preference analysis for ThBgl1 and ThBgl2. Finally, the analysis of the influence of temperature and pH on the activities of both enzymes are shown.

**Specifications Table**

**Table:** 

Subject area	*Biology*
More specific subject area	*Structural Enzymology*
Type of data	*Table and Figures*
How data was acquired	*X-ray diffractometer and plate reader*
Data format	*Analyzed*
Experimental factors	*N/A*
Experimental features	*X-ray data collected from flash frozen single crystals in a home source using CuKα radiation. Enzyme activity data was measured using the synthetic substrate p-nitrophenyl-glucopyranoside.*
Data source location	*São Carlos, SP, Brazil. -22.008911, -47.897772.*
Data accessibility	*Structural data is public through the protein data bank (PDB), with access codes 5JBK and 5JBO.*

**Value of the data**

• The data provided in the table shows the quality of the crystal structure used for the analysis of the mechanism of transglycosylation observed in these GH1 β-glucosidases.
• The substrate preference data provided shows that, although the enzymes are very similar, they have marked differences in substrate preference.
• The influence of pH and temperature indicate the optimal conditions for catalysis for the enzymes ThBgl1 and ThBgl2.

## 1 Data

Three sets of data are shown. First, the statistics and parameters from the X-ray diffraction data processing and structural refinement are given for the crystal structures of the enzymes ThBgl1 and ThBgl2. Second, the preferences for different natural and synthetic substrates are shown for these enzymes and, finally, the influence of pH and temperature on the enzyme activity is provided.

## 2 Experimental design, materials and methods

**Fig. 1:**
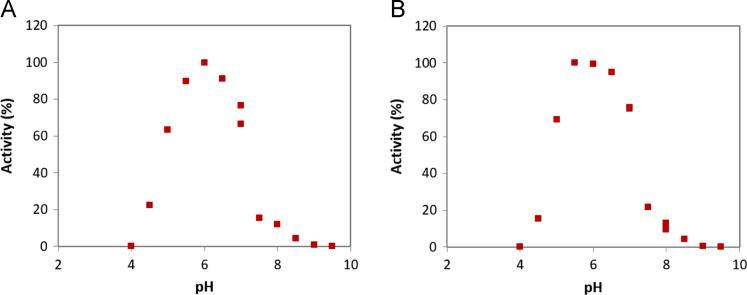
Influence of pH on ThBgl1 (A) and ThBgl2 (B) activities in *p*NPG.

**Fig. 2:**
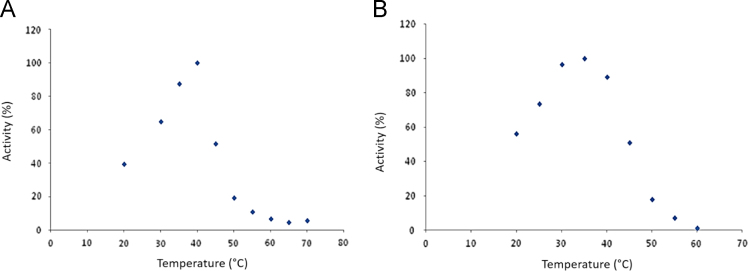
Temperature influence on ThBgl1 (a) and ThBgl2 (b) activities measured in pNPG.

The complete description of the methods is found in the associated research article [1]. For the determination of optimum pH and temperatures for enzyme activity, a reaction mixture using the synthetic substrate 4-nitrophenyl-β-D-glucopyranoside (*p*NPG Fig. 1). Briefly, 50 μL of *p*NPG (final concentration of 5 mM), 40 μL of 150 mM citrate-phosphate or phosphate buffer at different pHs and 10 μL of enzyme at 0.1 mg/ml were incubated for 5, 10, 15 and 20 minutes at 30 °C. The reaction was stopped by adding 100 μL of Na_2_CO_3_ 0.5 M and the amount of released products was measured spectrophometrically at 415 nm. For the determination of optimum temperature, the same reactions were incubated in a temperature range spanning 20 to 70 °C in 5 °C steps (Fig. 2). After 10 minutes, the reaction was stopped by adding 100 μL of Na_2_CO_3_ 1 M and the amount of released products was measured spectrophometrically at 405 nm.

The substrate preferences for ThBgl1 and ThBgl2 were evaluated using different synthetic substrates: *p*NPG, 4-nitrophenyl-β-D-xylopyranoside (*p*PNX), 4-nitrophenyl-α-D-galactopyranoside (*p*NPGal), 2-nitrophenyl-β-D-galactopyranoside, 4-nitrophenyl-β-D-cellobioside and 4-nitrophenyl-β-D-mannopyranoside (*p*NPM). All the reactions were tested at 35 °C for ThBgl1 and 40 °C for ThBgl2, using sodium phosphate buffer pH 5.5.

Purified ThBgl1 and ThBgl2 were used for crystallization trials at 25 mg/ml 30 mg/ml, respectively. For ThBgl1 crystals grew in 0.2 M hexahydrated magnesium chloride, 0.1 M HEPES pH 7.5 and 25% PEG3350. For ThBgl2, suitable crystals grew in a solution containing 0.1 MES pH 6.5, 25% PEG 8000. The crystals were flash frozen in a nitrogen stream under 100 K and used for data collection. For ThBgl1 crystal a complete dataset was collected in the MX-2 beamline of the Brazilian Synchrotron Light Source [2]. For ThBgl2 a complete dataset was collected using a Bruker APEX-Duo home source using copper Kα radiation. The scattered intensities were integrated using iMosflm [3] and, after scaling with AIMLESS [4], the structure factors were used for phasing by molecular replacement using the crystal structure of *T. reesei* GH1 β-glucosidase as the search model [5] and PHASER [6] software. Finally, the crystal structures were refined in iterative cycle of real space manual refinement using Coot [7] and reciprocal space refinement using PHENIX [8] software (Table 1).

**Table 1:** Data collection and refinement statistics.

Parameters	ThBgl1	ThBgl2
PDB code	5JBK	5JBO
Wavelength (Å)	1.46	1.54
Resolution range (Å)	71.92–2.59 (2.69–2.59)	61.64–1.97 (2.04–1.97)
Space Group	P 21 21 21	P 21 21 21
Unit cell	94.9 97.7 106.2	57.5 78.1 100.3
	90 90 90	90 90 90
Total reflections	31,180 (3015)	32,663 (3212)
Multiplicity	2.2	4.5
Completeness (%)	99.7 (97.6)	99.9 (100.0)
Mean I/sigma(I)	8.3 (2.2)	8.7 (3.1)
Wilson B-factor (Å^2^)	17.46	9.23
R_merge_	0.54	0.50
R_work_	0.213 (0.271)	0.1681 (0.2193)
R_free_	0.254 (0.308)	0.2025 (0.2640)
Number of non-hydrogen atoms	7973	4664
Macromolecules	7463	3797
Water	498	867
Ligands	12	0
Protein residues	930	475
RMS (_bonds_) (Å)	0.005	0.004
RMS (_angles_) (°)	1.06	1.03
Ramachandran favoured (%)	96	97
Ramachandran allowed (%)	4	3
Ramachandran outliers (%)	0	0
*Clashscore*	6.25	2.59
Average B-factor	16.40	11.80
Macromolecules	16.30	9.20
Ligands	16.40	0
Solvent	18.10	23.20
